# Causal relationship between gut microbiota and type 2 diabetes: a two-sample Mendelian randomization study

**DOI:** 10.3389/fmicb.2023.1184734

**Published:** 2023-08-24

**Authors:** Hanjing Li, Candong Li

**Affiliations:** ^1^College of Traditional Chinese Medicine, Fujian University of Traditional Chinese Medicine, Fuzhou, Fujian, China; ^2^Research Base of Traditional Chinese Medicine Syndrome, Fujian University of Traditional Chinese Medicine, Fuzhou, Fujian, China; ^3^Key Laboratory of Traditional Chinese Medicine Health Status Identification, Fuzhou, Fujian, China

**Keywords:** causal relationship, gut microbiota, genome-wide association study, Mendelian randomization, type 2 diabetes

## Abstract

**Background:**

Studies showed that development of gut microbial dysbiosis has a close association with type 2 diabetes (T2D). It is not yet clear if there is a causal relationship between gut microbiota and T2D.

**Methods:**

The data collected from the published genome-wide association studies (GWASs) on gut microbiota and T2D were analyzed. Two-sample Mendelian randomization (MR) analyses were performed to identify causal relationship between bacterial taxa and T2D. Significant bacterial taxa were further analyzed. To confirm the findings’ robustness, we performed sensitivity, heterogeneity, and pleiotropy analyses. A reverse MR analysis was also performed to check for potential reverse causation.

**Results:**

By combining the findings of all the MR steps, we identified six causal bacterial taxa, namely, *Lachnoclostridium, Oscillospira, Roseburia, Ruminococcaceae UCG003, Ruminococcaceae UCG010* and *Streptococcus*. The risk of T2D might be positively associated with a high relative abundance of *Lachnoclostridium, Roseburia* and *Streptococcus* but negatively associated with *Oscillospira, Ruminococcaceae UCG003* and *Ruminococcaceae UCG010.* The results of MR analyses revealed that there were causal relationships between the six different genera and T2D. And the reverse MR analysis did not reveal any evidence of a reverse causality.

**Conclusion:**

This study implied that *Lachnoclostridium, Roseburia* and *Streptococcus* might have anti-protective effect on T2D, whereas *Oscillospira*, *Ruminococcaceae UCG003* and *Ruminococcaceae UCG010* genera might have protective effect on T2D. Our study revealed that there was a causal relationship between specific gut microbiota genera and T2D.

## Introduction

Type 2 diabetes (T2D) is typically diagnosed in older individuals or middle-aged people. It is a metabolic disorder that can be caused by either beta cell dysfunction or insulin resistance (Taylor, 2013). The evidence supporting the increasing prevalence of T2D has shown a steady rise over the past few decades (Lastra et al., 2014). The rapid emergence and prevalence of diabetes has attracted global attention (Jaacks et al., 2016). In 2021, International Diabetes Federation released that the global diabetes prevalence rate among 20-79-year-old people is expected to increase to 12.2% in 2045, and most of them will be T2D (Sun et al., 2022).

The development of T2D can be triggered by various factors, such as unhealthy lifestyle and genetic factors. It has been theorized that the composition of gut microbiome plays a role in pathogenic mechanism of T2D (Cani, 2018; Hsieh et al., 2018; Zaky et al., 2021; Liu et al., 2022). Recently, great interest has been attracted by the gut microbiota which have been demonstrated in studies regulating host physiological activities. Studies have shown that gut microbiome were important in maintaining homeostasis (Geva-Zatorsky et al., 2017) and the establishment of immune system (Shi et al., 2017). Gut microbiota have important roles in the pathological process for various diseases by affecting the cell differentiation (Jubair et al., 2018) and cytokine release(Chappert, 2014), as well as regulating drug absorption and metabolism (Scher et al., 2020). Gut microbiota in individuals with T2D differs from non-diabetic ones (Larsen et al., 2010; Kashtanova et al., 2018; Takagi et al., 2020), the development of gut microbial dysbiosis has known to be a clinical manifestation of T2D (Forslund et al., 2015; Wu et al., 2017). But the results of various studies differ from one another. Larsen’s study indicated that the proportion of Firmicutes was significantly higher in healthy individuals compared with the diabetic, Bacteroidetes was somewhat enriched in the diabetic group, which results in a lower Firmicutes/Bacteroidetes ratio (Larsen et al., 2010). Nevertheless, Sedighi et al. found that an increased level of Firmicutes and decreased level of Bacteroidetes in T2D individuals, which means a higher Firmicutes/Bacteroidetes ratio in diabetic group (Sedighi et al., 2017). Whilst the specific microbiome features identified as responsible for the appearance of T2D have differed among studies (Doumatey et al., 2020; Kitten et al., 2021), Kootte’s study showed that insulin sensitivity increased in obese metabolic syndrome participants who received fecal microbiota transplantation (FMT) from lean donors (Kootte et al., 2017). As a valid treatment for patients with T2D, FMT introduced (transplanted) gut microbiota acquired from the faeces of healthy donors into the patient’s gastrointestinal tract (Antushevich, 2020). This is also a good application of gut microbiota in the treatment of T2D. The most important intention for research is to provide guidance for microbiota-orientated interventions treating T2D clinically.

Although the correlation between the gut microbiome and T2D is widely acknowledged, the exact causal relationship remains unclear. Elucidation of the causal relationships could be helpful for deepening comprehending of the responsibility of gut microbiota for the pathogenesis of T2D. As a statistical method, Mendelian randomization (MR) is usually used to clarify causal relationship between exposures to outcomes. MR uses single-nucleotide polymorphisms (SNPs) which strongly related to exposure as instrumental variables (IVs), and the relationship between the exposure and outcome could be evaluated (Emdin et al., 2017). The results of large-scale genome-wide association studies (GWASs) of the gut microbiota and diseases have led to the widespread use of MR analysis in various research fields (Bonder et al., 2016; Goodrich et al., 2016). Unlike traditional observational studies, MR assists researchers in identifying the causal relationship between the exposure and outcome directly, and interference of reverse causation and confounding factors can be avoided (Zheng et al., 2017). Our research performed a two-sample MR analysis to explore relationship between gut microbiota and T2D.

## Materials and methods

### Ethics statement

**Figure 1:**
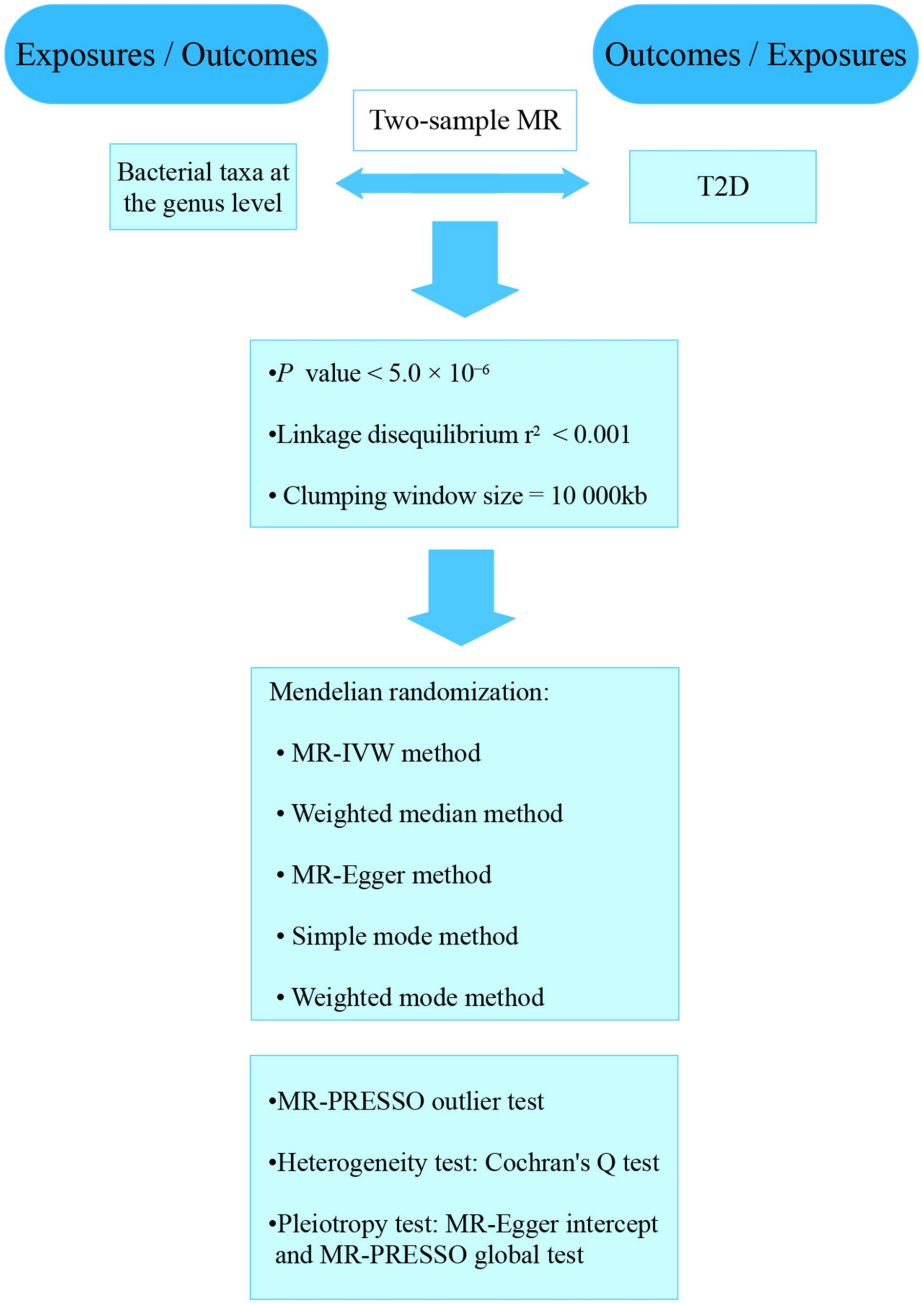
The flowchart of the study.

Our analyses used the summary statistics of publicly available GWASs. No new data was collected, and the study was conducted without new ethical approval. The whole process that we studied was presented in a flowchart in Figure 1. The gut microbiota was the exposure, and T2D was outcome. A reverse MR analysis was also performed to examine the effects of T2D on the gut microbiota.

### Gut microbiota sample

The summary statistics data collected from a large-scale GWAS meta-analysis which analyze the gut microbial taxa of 18,340 participants from various ethnic groups (Kurilshikov et al., 2021). The twenty cohort studies included single ancestry samples from various regions, such as European (*n* = 13,266), Middle Eastern (*n* = 481), Latin American (*n* = 1,097), East Asian (*n* = 811), African American (*n* = 114). The data collected from four cohorts included multiple ancestries (*n* = 2,571). The researchers analyzed the microbial composition using seven different extraction methods and three different rRNA regions. All the datasets were rarefied to around 10,000 reads per sample to be obligated to sequencing depth differences. Only taxa that belonged to over 10% of all the samples were included in this study to examine the effects of host genetic variation on gut bacterial species. The researchers analyzed 211 taxa. The total included 131 genera, 35 families and 20 orders, as well as 16 classes, 9 phyla.

### T2D sample

GWAS summary statistics for T2D were gathered from a large sample meta-analysis from the publicly available GWAS analyses. The study investigated T2D cases (62,892) and controls (596,424), which included over 16 million genetic variants by combining three GWAS datasets of European (Xue et al., 2018).

### Selection of IVs

The total bacterial taxa included 211 species at five taxonomic levels. We analyzed with genus level which is the smallest and most specific of above illustrated levels with the current 16S rRNA gene sequencing technology. We analyzed 131 taxa and excluded 12 unknown group taxa. Then, we included 119 bacterial taxa in the subsequent MR analyses.

To select potential IVs, we first included SNPs those associated with gut bacterial taxa at the genome-wide significance threshold *p* < 5.0 × 10^−6^. Eligible IVs must meet the followed quality control steps. First, the linkage disequilibrium (LD) threshold for clumping was set to *r*^2^ < 0.001 and the window size for clumping 10,000 kb to minimize the effects of LD on the results. Second, we selected only exposures those had at least three independent genetic instruments. Third, we excluded palindromic SNPs with middle allele frequency. We calculated the *F* statistic based on the formula: *F* = (β/SE)^2^.

### Statistical analysis

Effects of gut microbiota on T2D were estimated through a multi-test MR analysis. For the bacterial genera, various tests were performed included the random-effects inverse variance weighted (IVW) (Burgess et al., 2013), MR-Egger regression and weighted median. The evaluation of the heterogeneity related to each bacterial genus was conducted by the Cochrane’s Q statistics. The random-effects IVW test was used to make sure a conservative but robust estimate when heterogeneity existed (*p* < 0.05). The weighted median test can also provide consistent estimates if more than 50% of the weights come from valid IVs (Bowden et al., 2016). The MR-Egger regression allows for the presence of pleiotropy in more than 50% of IVs (Bowden et al., 2015). Further analyses were conducted on bacterial genera that were identified to be significant in the previous steps. With MR-Egger regression, we detected horizontal pleiotropy. Based on the “leave-one-out” method, we conducted sensitivity analysis (Dong et al., 2023).

### Reverse MR analysis

We carried out reverse MR analysis to examine the effect of T2D on the identified bacterial genus. SNPs related to T2D were used as IVs. The results of the various statistical analyses were generated using R (version 4.2.2). The “TwoSampleMR” package was applied to perform IVW, weighted median, and MR-Egger regression. Through the “MRPRESSO” package, we conducted MR-PRESSO test.

## Results

### Selection of IVs

According to the selection criteria of IVs, 482 SNPs associated with 97 genera were selected as IVs. F statistics for all SNPs were greater than 10, which suggested there were no weak IVs (Supplementary Table S1).

### Causal effects of gut microbiota on T2D

Of all the 97 genera, six significant bacterial genera were selected for further MR analyses. Furthermore, four independent SNPs were associated with *Lachnoclostridium* and *Oscillospira*, respectively. Five independent SNPs were associated with *Ruminococcaceae UCG010*, eight SNPs were associated with *Roseburia* and *Streptococcus*. Nine SNPs were associated with *Ruminococcaceae UCG003* (Supplementary Table S2). SNP detailed message (Position, OR, Effect, Function) of significant genera in MR analyses were shown in Table 1.

**Table 1:** SNP message of significant genera.

Bacterial taxa (exposure)	SNP	Position	OR	Effect	Function
Lachnoclostridium	rs4738679	chr8:59370320	1.009	Anti-protective effect	Intergenic
	rs6112314	chr20:19300846	0.994	Protective effect	Intron
	rs789029	chr18:1053252	0.989	protective effect	Intron
	rs615997	chr3:23037786	1.011	Anti-protective effect	Downstream
Oscillospira	rs12206468	chr6:18093691	1.007	Anti-protective effect	Intergenic
	rs28889936	chr4:89483300	0.977	Protective effect	Intron
	rs1954532	chr14:28151415	0.974	Protective effect	Intron
Roseburia	rs16910295	chr11:12009569	0.999	Protective effect	Intron
	rs9300744	chr13:103117486	0.984	Protective effect	Intergenic
	rs6445851	chr3:57116228	1.024	Anti-protective effect	Upstream
	rs6930661	chr6:12774611	0.999	Protective effect	Intron
	rs2160994	chr12:50650057	0.990	Protective effect	Intron
	rs2943022	chr5:89598914	1.004	Anti-protective effect	intergenic
Ruminococcaceae UCG003	rs73341549	chr7:51541468	1.009	Anti-protective effect	Intergenic
	rs16959793	chr15:35071718	1.016	Anti-protective effect	Intron
	rs3013089	chr1:13794594	1.007	Anti-protective effect	Intergenic
	rs4532474	chr6:105781538	1.001	Anti-protective effect	Intron
	rs11243416	chr9:134416970	0.998	Protective effect	Upstream
	rs4452755	chr8:82026852	1.011	Anti-protective effect	Upstream
	rs11613919	chr12:75496463	0.992	Protective effect	Intron
	rs10490280	chr2:37905976	1.011	Anti-protective effect	Intron
	rs646327	chr19:49209851	0.986	Protective effect	Downstream
Ruminococcaceae UCG010	rs2820282	chr6:104728618	0.991	protective effect	Intergenic
	rs12597105	chr16:5233941	1.010	Anti-protective effect	Intergenic
	rs6958419	chr7:16349864	1.016	Anti-protective effect	Intron
	rs682403	chr9:135968557	1.015	Anti-protective effect	Downstream
Streptococcus	rs11720390	chr3:94103591	1.031	Anti-protective effect	Intergenic
	rs7916711	chr10:28588269	0.998	Protective effect	Intron
	rs10448310	chr9:93556174	0.985	Protective effect	Intergenic
	rs6806351	chr3:132058723	1.009	Anti-protective effect	Intron
	rs4968759	chr17:61298020	0.995	Protective effect	Intron
	rs11764382	chr7:46774896	0.974	Protective effect	Intron
	rs17708276	chr8:10199548	0.973	Protective effect	Intron
	rs11110281	chr12:100584014	0.979	Protective effect	Intron

**Figure 2:**
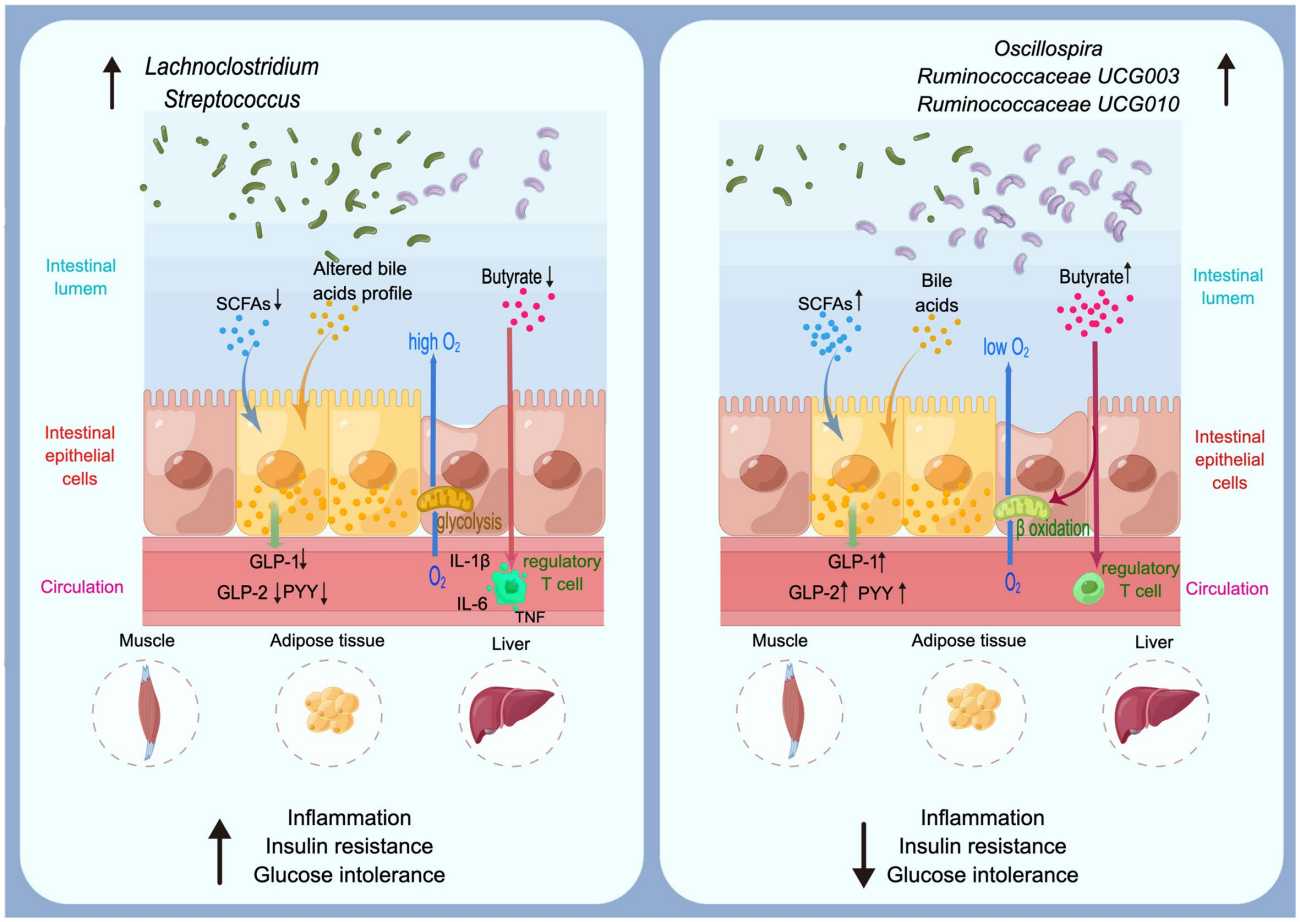
The underlying mechanisms of significant genera on type 2 diabetes.

The genetically predicted relative abundances of six genera were associated with T2D. Indeed, high genetically predicted levels of *Oscillospira* (OR: 0.834, 95% CI: 0.712–0.976), *Ruminococcaceae UCG003* (OR: 0.885, 95% CI: 0.811–0.967) and *Ruminococcaceae UCG010* (OR: 0.812, 95% CI: 0.713–0.925) were negatively associated with the risk of T2D. Nevertheless, *Lachnoclostridium* (OR: 1.175, 95% CI: 1.011–1.366), *Roseburia* (OR: 1.192, 95% CI: 1.033–1.374) and *Streptococcus* (OR: 1.199, 95% CI: 1.600–1.357) were positively associated with the risk of T2D. The weighted median method also supported parts of the results. The genetically predicted *Streptococcus* level was positively associated with the risk of T2D (OR: 1.211, 95% CI: 1.051–1.395). Meanwhile, *Oscillospira* (OR: 0.840, 95% CI: 0.707–0.998), *Ruminococcaceae UCG003* (OR: 0.888, 95% CI: 0.791–0.996) and *Ruminococcaceae UCG010* (OR: 0.823, 95% CI: 0.700–0.967) might have protective effect on T2D (Tables 2, 3). The underlying mechanisms of significant genera on T2D were shown in Figure 2.

**Table 2:** Significant MR analysis results.

Bacterial taxa (exposure)	MR method	No. SNP	OR	95% Cl	*p*
Lachnoclostridium	IVW	4	1.175	1.011–1.366	0.036
	Weighted median		1.182	0.995–1.405	0.058
	MR-Egger		1.023	0.167–6.285	0.982
	Simple mode		1.190	0.953–1.486	0.223
	Weighted mode		1.191	0.945–1.500	0.235
Oscillospira	IVW	3	0.834	0.712–0.976	0.024
	Weighted median		0.840	0.707–0.998	0.048
	MR-Egger		1.391	0.719–2.691	0.506
	Simple mode		0.790	0.619–1.009	0.200
	Weighted mode		0.878	0.680–1.132	0.421
Roseburia	IVW	6	1.192	1.033–1.374	0.016
	Weighted median		1.128	0.950–1.339	0.169
	MR-Egger		0.772	0.464–1.284	0.376
	Simple mode		1.089	0.866–1.369	0.498
	Weighted mode		1.089	0.854–1.388	0.523
Ruminococcaceae UCG003	IVW	9	0.885	0.811–0.967	0.007
	Weighted median		0.888	0.791–0.996	0.043
	MR-Egger		1.091	0.805–1.480	0.591
	Simple mode		0.860	0.709–1.045	0.167
	Weighted mode		0.879	0.736–1.049	0.190
Ruminococcaceae UCG010	IVW	4	0.812	0.713–0.925	0.002
	Weighted median		0.823	0.700–0.967	0.018
	MR-Egger		1.533	0.145–16.195	0.756
	Simple mode		0.851	0.693–1.045	0.221
	Weighted mode		0.852	0.701–1.037	0.208
Streptococcus	IVW	8	1.199	1.600–1.357	0.004
	Weighted median		1.211	1.051–1.395	0.008
	MR-Egger		1.211	0.797–1.841	0.404
	Simple mode		1.344	1.065–1.695	0.041
	Weighted mode		1.300	1.017–1.661	0.074

**Table 3:** Significant genera effect on T2D.

Bacterial taxa	Effect	Potential contributes
Lachnoclostridium	Anti-protective effect	Pro-inflammation
Oscillospira	Protective effect	Anti-inflammation, maintain the gut barrier
Roseburia	Anti-protective effect	Pro-inflammation
Ruminococcaceae UCG003	Protective effect	Anti-inflammation, maintain the gut barrier
Ruminococcaceae UCG010	Protective effect	Anti-inflammation, maintain the gut barrier
Streptococcus	Anti-protective effect	Pro-inflammation

### Sensitivity analyses

**Figure 3:**
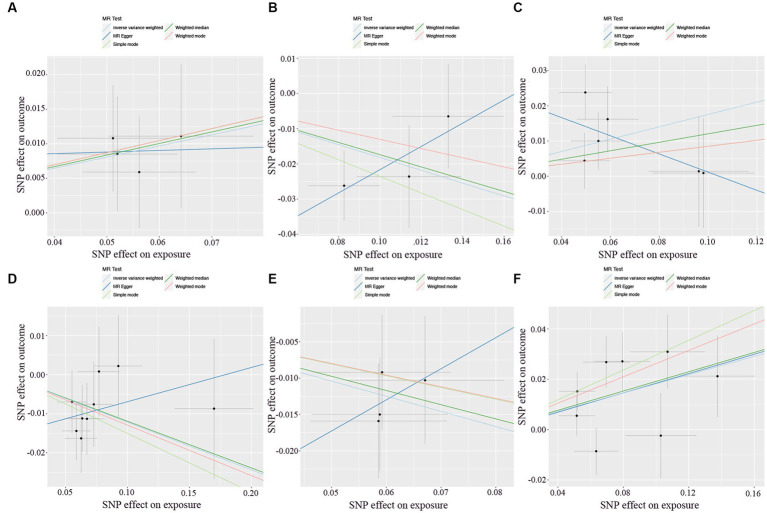
Scatter plots of each genus associated with the risk of type 2 diabetes. **(A)** *Lachnoclostridium*. **(B)** *Oscillospira*. **(C)** *Roseburia*. **(D)** *Ruminococcaceae UCG003*. **(E)** *Ruminococcaceae UCG010*. **(F)** *Streptococcus*.

**Figure 4:**
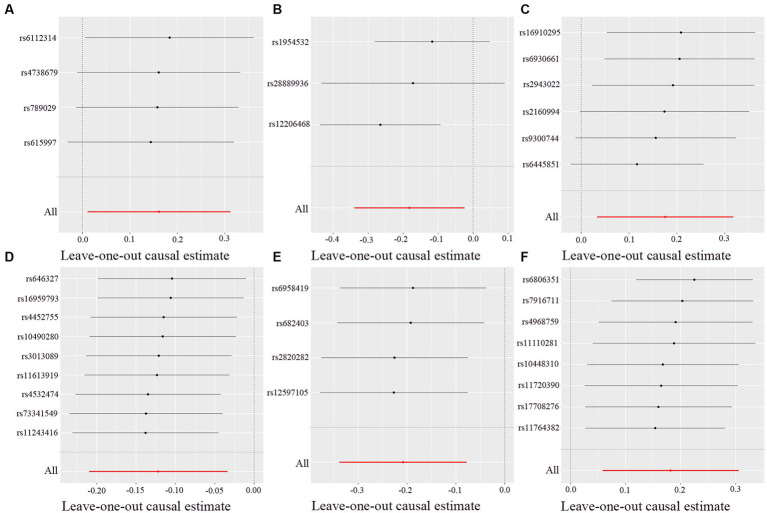
Leave-one-out analysis of each genus associated with type 2 diabetes. **(A)** *Lachnoclostridium*. **(B)** *Oscillospira*. **(C)** *Roseburia*. **(D)** *Ruminococcaceae UCG003*. **(E)** *Ruminococcaceae UCG010*. **(F)** *Streptococcus*. Red lines represent estimations from the IVW test.

There was no heterogeneity (A genetic trait can be caused by changes in multiple different genetic materials) within the IVs of all the six genera (Supplementary Table S3). The MR-Egger regression intercepts indicated no horizontal pleiotropy (*p* > 0.05) (Supplementary Table S4). The scatter plots illustrated that *Oscillospira, Ruminococcaceae UCG003 and Ruminococcaceae UCG010* genera might have protective effect on T2D, whereas *Lachnoclostridium, Roseburia and Streptococcus* genera might have anti-protective effect on T2D. The weights of MR analysis methods described in the scatter plots are listed in order, the IVW method, the MR-Egger, weighted median, weighted mode, and simple mode. The lines moving upward from left to right were found to be positive indicators of the relationship between the genus and T2D, while those going down from left to right were protective genera (Figure 3). There were no potential outliers of the IVs of all six genera for T2D in “leave-one-out” analysis, suggesting that all the identified causal associations were not influenced by single IV (Figure 4). Reverse MR results analysis revealed that T2D had no causal impact on all six genera (Table 4).

**Table 4:** Reverse causal association between T2D and gut microbiota.

Bacterial taxa (outcome)	MR method	No. SNP	OR	95% Cl	*p*
Lachnoclostridium	IVW	108	1.022	0.990–1.054	0.186
	Weighted median		1.008	0.956–1.064	0.770
	MR-Egger		1.008	0.938–1.083	0.832
	Simple mode		1.042	0.933–1.164	0.497
	Weighted mode		1.026	0.964–1.091	0.436
Oscillospira	IVW	108	0.995	0.955–1.038	0.827
	Weighted median		1.059	0.989–1.135	0.120
	MR-Egger		1.083	0.986–1.191	0.100
	Simple mode		1.058	0.897–1.248	0.504
	Weighted mode		1.066	0.971–1.171	0.133
Roseburia	IVW	108	1.024	0.992–1.057	0.139
	Weighted median		1.043	0.988–1.100	0.124
	MR-Egger		1.025	0.954–1.101	0.496
	Simple mode		0.944	0.849–1.051	0.327
	Weighted mode		1.027	0.965–1.093	0.407
Ruminococcaceae UCG003	IVW	108	1.034	0.992–1.077	0.107
	Weighted median		1.037	0.969–1.111	0.304
	MR-Egger		1.074	0.980–1.178	0.131
	Simple mode		1.077	0.954–1.215	0.246
	Weighted mode		1.040	0.966–1.119	0.240
Ruminococcaceae UCG010	IVW	108	1.005	0.967–1.045	0.754
	Weighted median		0.993	0.920–1.072	0.853
	MR-Egger		1.016	0.929–1.110	0.699
	Simple mode		1.022	0.889–1.175	0.848
	Weighted mode		1.001	0.929–1.078	0.924
Streptococcus	IVW	108	0.984	0.952–1.017	0.332
	Weighted median		0.969	0.916–1.025	0.280
	MR-Egger		0.947	0.879–1.021	0.159
	Simple mode		1.078	0.945–1.228	0.236
	Weighted mode		0.964	0.903–1.029	0.321

## Discussion

Using two-sample MR methods, the causal relationship between the gut microbiota and T2D was clarified. By combining evidence from MR and sensitivity analyses, we identified that the bacterial genera *Lachnoclostridium, Oscillospira, Roseburia, Ruminococcaceae UCG003, Ruminococcaceae UCG010* and *Streptococcus* were causally associated with T2D. Among all six genera, *Lachnoclostridium, Roseburia* and *Streptococcus* were positively associated with the risk of T2D, they might have anti-protective effect on T2D. *Oscillospira, Ruminococcaceae UCG003* and *Ruminococcaceae UCG010* were negatively associated with the risk of T2D, in other words, the three genera might have protective effect on T2D.

The pathogeneses of T2D are controversial and only partly understood, in which gut microbiota might be included in several underlying mechanisms (Figure 2). Short-chain fatty acids (SCFAs) and bile acids (BAs) could activate several key receptors expressed by enteroendocrine cells, thus increasing the secretion of key gut peptides including glucagon-like peptide (GLP)-1, GLP-2, and peptide YY (PYY). GLP-1, GLP-2, and PYY lower glucose concentrations (Meier, 2012), intestinal permeability (Cani et al., 2009), cytokine and amylase release (Vona-Davis and McFadden, 2007), thereby improving metabolic disorders and inflammation during diabetes. Butyrate has been described in numerous studies which provide essential energy for proliferating of colonic cells and maintain the gut barrier (Martin-Gallausiaux et al., 2021; Stoeva et al., 2021; Tang et al., 2022). Indeed, butyrate contributes to control of the anaerobic condition in the colon which is a key requirement for the remain of anaerobic bacteria in the close vicinity of the epithelium through activating the β-oxidation in the mitochondria (Rardin et al., 2013). A reduce of butyrate-producing bacteria might active immune cells including T cells, which secrete pro-inflammatory cytokines including IL-1β, IL-6 and TNF (Castoldi et al., 2015; Li et al., 2021). Through these underlying mechanisms, gut microbiota is associated with T2D development by regulate numerous metabolic pathways in the gut and at distance such as in the muscles, the adipose tissue, and the liver.

Bacterial genera *Oscillospira, Ruminococcaceae UCG003* and *Ruminococcaceae UCG010* are all members of family Ruminococcaceae, phylum Firmicutes. Gut microbiota affected the major human diseases, such as obesity and diabetes, through metabolic and immune signals that enter the circulation (Holmes et al., 2012). Gut microbiota involving genera from family Ruminococcaceae can affect the production of SCFAs and the conversion of primary BAs to secondary BAs (Koh et al., 2016). SCFAs are important metabolites in maintaining intestinal homeostasis among which butyrate has been investigated most extensively (Parada Venegas et al., 2019). Gut microbiota could reshape the host metabolic, signaling pathways and intestinal barrier functions, which are related to the insulin resistance in T2D (Sharma and Tripathi, 2019). As a member of family Ruminococcaceae, *Oscillospira* is a genus capable of producing SCFAs such as butyrate. Studies have shown that the variation in *Oscillospira* abundance has a obviously favorable influence on human health (Konikoff and Gophna, 2016) especially for metabolic diseases (Konikoff and Gophna, 2016; Gophna et al., 2017) and it has been referred to as a potential next-generation probiotic (Yang et al., 2021). Through over 6.2 years of follow-up for 2,731 participants without T2D initially, Miao et al. found that compared with 276 diabetics, *Oscillospira* were enriched in participants without T2D (Miao et al., 2020). Shen et al. investigated the effect of L-arabinose for preventing or treating T2D, and they found that L-arabinose reversed the decrease in the relative abundance of *Oscillospira* in T2D rats as well as aggravation symptoms of diabetes mellitus (Shen et al., 2021). These results are evidence of the protective effect of *Oscillospira* on T2D. Butyrate-producing bacteria produce butyrate by fermenting carbohydrate. Accumulating evidences have shown that the butyrate can interact with various physiological function, such as glucose homeostasis and appetite (Canfora et al., 2015). Butyrate has important functions for host health (McMurdie et al., 2022); studies have illustrated that butyrate-producing bacteria can protect against various types of metabolic diseases (Qin et al., 2012; Valles-Colomer et al., 2019; Stoeva et al., 2021; McMurdie et al., 2022). Depletion in butyrate-producing taxa has been linked to the development of T2D (Qin et al., 2012). Although *Roseburia* is a major genus of butyrate-producing bacteria in the intestinal tract of animals and humans, we found it might be risk factor for T2D. *Roseburia* is also a member of phylum Firmicutes, the various species of *Roseburia* that are all known to produce SCFAs (Tamanai-Shacoori et al., 2017). Some works observed that *Roseburia* exerts beneficial effects on human health through producing metabolites to prevent intestinal inflammation and maintain energy homeostasis (Tamanai-Shacoori et al., 2017; Song et al., 2021). A metagenome-wide association study(MGWAS) discovered that patients with T2D showed a decreasing trend of *R. intestinalis* (Qin et al., 2012), which was different from our study.

*Ruminococcaceae UCG003* and *Ruminococcaceae UCG010* are two bacterial genera in the same family. Recent studies discovered that *Ruminococcaceae UCG003* is one of the main genera that plays a positive role in chronic insomnia and cardiometabolic diseases (Jiang et al., 2022), as well as depressive symptoms (Radjabzadeh et al., 2022). Huang et al. (2022) characterized the change in the gut microbiota composition after metabolic surgery in patients with diabetes. They found that the abundance of the *Ruminococcaceae UCG003* group increased after metabolic surgery (Huang et al., 2022), implying that the abundance of *Ruminococcaceae UCG003* was negatively associated with diabetes. In another study (Gao et al., 2020), levels of *Ruminococcaceae UCG003* abundance were observed significantly reduced in women with hyperglycemia in pregnancy. Our findings also support the result that *Ruminococcaceae UCG003* has protective effect against diabetes. Chen et al. (2021) performed a microbiome-wide study with a large population to examine the role of gut microbiome composition in insulin resistance as well as T2D. They found 12 groups of bacteria were related to T2D, which included *Ruminococcaceae UCG010*, and a high abundance of *Ruminococcaceae UCG010* was negatively associated with the risk of T2D, suggesting its protective effects against T2D. Differ from the above study, Esquivel-Hernández et al. observed that *Ruminococcaceae UCG010* is one of the genera which driving the progress of T2D in a Mexican cohort (Esquivel-Hernández et al., 2023).

A case–control study conducted by Allin et al. (2018) analyzed the gut microbiota between normal and anormal glucose regulation individuals, and they found that the abundance of various bacterial genera and over 30 operational taxonomic units (OTUs) were differentially between the two groups. In their findings, the abundance of *Streptococcus* increased in adults with prediabetes. Studies have shown that *Streptococcus* was closely related to various inflammation (Takahashi et al., 2011; Ge and Sun, 2014; Zbinden et al., 2015), which might be involved in the pathogeneses of T2D. Our findings also supported their results that increasing of *Streptococcus* abundance is a mark of progression to diabetes. Wang et al. (2020) observed the antiobesity effects of the natural polyphenol resveratrol (RSV) and found that RSV treatment significantly changed the composition of gut microbiome in mice, which showed an enrichment of six genera including *Lachnoclostridium*. Next, a high-fat diet treated mice were transplanted of the six RSV-microbiota, then the mice weight gain decreased, insulin sensitivity increased, and intestinal barrier function improved. Tettamanzi et al. (2021) conducted a 3-week randomized controlled crossover feeding trial to investigate the intervention mechanism of two types of dietaries including different components but isocaloric on 20 obese women with insulin-resistance. The study revealed that the genus *Lachnoclostridium* might be negatively impact to glucose metabolism, and thus, T2D. *Lachnoclostridium*, as a member of Lachnospiraceae, are also implied in BAs transformation (Thomas et al., 2008). Dysregulation of BAs homeostasis might associate with the progress of T2D (Chávez-Talavera et al., 2017; McGlone and Bloom, 2019). Our finding consistent with the result of Tettamanzi’s study, which suggested *Lachnoclostridium* was found to be positively correlated with T2D (Tettamanzi et al., 2021). Differ from our findings, Wang’s study showed that *Lachnoclostridium* had a positive effect on increasing insulin sensitivity (Wang et al., 2020).

Our study found a causal relationship between six genera of gut microbiota and T2D. Nevertheless, T2D is a multi-factorial disease that can be influenced by environment, gender, lifestyle, diet, aging, epigenetics, and genetics (Scott et al., 2007; Ling and Groop, 2009; NCD Risk Factor Collaboration, 2016). Previous studies have shown that the role of genera *Roseburia, Rumnococcaceae UCG010*, and *Lachnoclostridium* in T2D are controversial, and different studies have yielded different results. Our findings provide reference for dispute resolution. A change in an individual bacterium cannot account for the incidence of T2D, specific genus may have a different type of effect in specific biological context. Two large-scale metagenome analyses explored the structural features of gut microbiota in China and Europe healthy people and T2D patients, which illustrated that large differences still exist in metagenomic clusters of cohorts in both area (Qin et al., 2012; Karlsson et al., 2013). It shows that the susceptibility for T2D would not be affected by single bacteria. This situation maybe an explanation for the inconsistency of our results with some other findings.

Our study has several limitations. First, participants in the two GWASs were primarily of European descent, therefore the applicability of our results need further investigation. Second, current research methods of microbiota GWASs limit the scope of our study for further specialized level. The specificity and accuracy of current results can be increased by using advanced analyzing method, then the generalizability of our results and the accuracy of our study might be improved. Related work needs to be done to identify associations between T2D and gut microbiome by combining the evidence from cohort studies, clinical trials, and functional studies, such an investigation is conducive for exploring the pathogenesis of diabetes.

## Conclusion

Our study found that there could be causal implications for T2D from the presence of six genera in the gut microbiome. *Oscillospira, Ruminococcaceae UCG003,* and *Ruminococcaceae UCG010* were identified to be negatively associated with the risk of T2D. *Lachnoclostridium, Roseburia,* and *Streptococcus* might have anti-protective effect on T2D.

## Data availability statement

The datasets presented in this study can be found in online repositories. The names of the repository/repositories and accession number(s) can be found in the article/Supplementary material.

## Author contributions

HL designed the study, collected, and analyzed the data and wrote the manuscript. CL edited the manuscript. All authors contributed to the article and approved the submitted version.

## Funding

This study was funded by Joint Funds of the National Natural Science Foundation of China (No. U1705286).

## Conflict of interest

The authors declare that the research was conducted in the absence of any commercial or financial relationships that could be construed as a potential conflict of interest.

## Publisher’s note

All claims expressed in this article are solely those of the authors and do not necessarily represent those of their affiliated organizations, or those of the publisher, the editors and the reviewers. Any product that may be evaluated in this article, or claim that may be made by its manufacturer, is not guaranteed or endorsed by the publisher.
